# Letter to the Editor on AI-Driven CBCT Analysis for Surgical Decision by Deng et at

**DOI:** 10.1016/j.identj.2026.109544

**Published:** 2026-04-07

**Authors:** Yaojing Chen

**Affiliations:** Department of Stomatology, Tiantai People’s Hospital of Zhejiang Province (Tiantai Branch of Zhejiang Provincial People’s Hospital), Hangzhou Medical College, Taizhou, Zhejiang, China

Dear Editor,

I read with great interest the article by Deng et al titled 'AI-Driven CBCT Analysis for Surgical Decision-Making and Mucosal Damage Prediction in Sinus Lift Surgery for Patients with Low RBH', recently published in the International Dental Journal.^1^ The authors present a pioneering 3D deep-learning system integrating the Convolutional Block Attention Module and depth-wise separable convolutions into a 3D convolutional network for maxillary sinus floor elevation (MSFE) decision-making and Schneiderian membrane perforation prediction. This work represents a significant advancement beyond traditional experience-based planning. Below, I outline several key perspectives for further consideration.

## Spatial modelling, multimodal fusion, and clinical interpretability

The shift from 2D slice-based analysis to full 3D-CBCT volume processing represents a major step forward, enabling comprehensive capture of the complex three-dimensional anatomy of the maxillary sinus. The use of the Convolutional Block Attention Module allows the model to focus on anatomically relevant regions – such as sinus floor contour, lateral wall thickness, residual ridge height, and sinus membrane thickness^2^ – while Grad-CAM visualizations provide clinically meaningful explanations. Notably, the high spatial concordance between attention heatmaps and expert-annotated regions suggests that the model has learned to prioritize genuine anatomical features rather than spurious correlations.

However, surgical decision-making in MSFE is inherently multifactorial. In routine clinical practice, perforation risk is influenced not only by residual bone height, but also by lateral sinus wall thickness, sinus floor morphology, presence and orientation of sinus septa, intraosseous vascular anatomy, Schneiderian membrane thickness, and intrasinus pathology, as well as patient-related factors such as smoking status, diabetes control, and history of chronic sinusitis.^3^ Therefore, multimodal data fusion – combining CBCT imaging with structured clinical records, anatomical classifications, biomechanical simulation data (eg, finite element analysis of membrane stress distribution during elevation^4^), and intraoral scanning data – may substantially enrich feature representation and improve individualized risk stratification, ultimately enabling the model to reflect real-world surgical complexity more faithfully.

## Intraoperative dynamic navigation and real-time risk assessment

While the current model focuses on preoperative planning, its greatest translational potential may lie in intraoperative guidance. Integrating the AI model with real-time navigation systems could enable dynamic risk assessment during surgery. For instance, augmented reality overlays projecting high-risk perforation zones onto the surgical field could provide intuitive guidance for less experienced surgeons.^5^ Such real-time feedback would transform the system from a static preoperative planning tool into an adaptive intraoperative decision-support platform, substantially enhancing procedural safety.

## Multicentre validation and methodological considerations

The authors appropriately acknowledge the limitations of the single-centre retrospective design with a relatively modest sample size (*n* = 79). To improve model generalizability, future research should prioritize multicentre prospective studies incorporating CBCT datasets from diverse scanner brands and patient populations, including those with maxillary sinus pathologies. Given the interinstitutional variability in CBCT acquisition parameters (eg, field of view, voxel size, reconstruction algorithms), domain adaptation techniques or harmonization strategies should be explored to ensure consistent model performance. Federated learning approaches could further enable multicentre collaboration while preserving patient privacy.

Methodologically, the class imbalance between perforation and nonperforation cases (approximately 15% perforation rate) may have influenced model training; advanced techniques such as focal loss or ensemble methods could improve minority-class sensitivity. Furthermore, leveraging deep learning for automatic maxillary sinus segmentation on CBCT – as recently demonstrated^6^^,^^7^ – could facilitate automated extraction of anatomical parameters and pathology classification, improving standardization and scalability. Exploring newer architectures such as Vision Transformers adapted for 3D volumetric data may also yield further performance gains.

In summary, Deng et al have delivered a timely and methodologically sound contribution to AI-assisted oral surgery. Their innovative application of 3D CNN with attention mechanisms to MSFE decision-making marks a significant milestone. With continued multicentre validation and expansion towards multimodal, workflow-integrated systems, this promising technology has the potential to meaningfully enhance the safety, consistency, and precision of maxillary sinus lift procedures.

## Conflict of interest

The author declares that she has no known competing financial interests or personal relationships that could have appeared to influence the work reported in this article.
